# Knowledge, Awareness and Practice Regarding the Spread of Oro-Dental Infections to the Head and Neck Region: A Comparative Cross-Sectional Survey Among Dental and Medical Students

**DOI:** 10.7759/cureus.117271

**Published:** 2026-09-30

**Authors:** Nomaan A Shamlik, Ashwinirani SR, Girish Suragimath, Kamala K A, Vaibhavi S Patane, Saae S Patil

**Affiliations:** 1 Oral Medicine and Radiology, School of Dental Sciences, Krishna Vishwa Vidyapeeth (Deemed to be University), Karad, IND; 2 Periodontology, School of Dental Sciences, Krishna Vishwa Vidyapeeth (Deemed to be University), Karad, IND

**Keywords:** amoxicillin, head and neck infections, knowledge attitude and practice, ludwig's angina, odontogenic infection

## Abstract

Introduction

Odontogenic infections can spread along fascial planes to the head and neck region, occasionally progressing to life-threatening complications such as Ludwig's angina. Since Medical and Dental students are future healthcare professionals who will encounter such conditions, assessing their comparative knowledge, awareness, and practice is essential for targeted educational improvement.

Methodology

A structured questionnaire-based cross-sectional survey was administered online to 200 students - 100 Dental (BDS) and 100 Medical (MBBS) - across various academic years to assess and compare knowledge, awareness, and practice regarding oro-dental infections and their head-and-neck complications. Data were statistically analyzed using the Chi-square test.

Results

Dental students consistently demonstrated higher awareness than Medical students across most domains, including facial and cervical spread of infection, lymph node involvement, and the danger of untreated infection. They also sought treatment earlier and identified management protocols more accurately, with 68% correctly identifying amoxicillin as the first-line antibiotic versus 51% among Medical students, and 58% recognizing airway obstruction as the most serious risk in Ludwig's angina compared to 49%. Not all group differences reached statistical significance on chi-square testing; the strongest differences (untreated-infection danger, management knowledge, early-consultation belief) were highly significant (p < 0.001), while several others, including antibiotic selection and Ludwig's angina risk recognition, did not reach significance once corrected for chance.

Conclusion

Dental students demonstrated higher knowledge, awareness, and more favorable practice than Medical students in several domains, such as management knowledge, treatment-seeking behavior, and perceived severity of infection, while other domains, including antibiotic selection and Ludwig's angina risk recognition, showed no significant difference between groups. Strengthening interdisciplinary curricula and structured clinical exposure is recommended to improve early recognition and timely referral for both disciplines.

## Introduction

Odontogenic infections arise from dental caries, periapical pathology, or periodontal disease and represent a significant source of morbidity in both dental and medical practice [1]. When inadequately treated, these infections can traverse anatomical tissue planes involving the submandibular, sublingual, parapharyngeal, and other adjacent deep spaces, leading to complications including mediastinitis, septicemia, and life-threatening airway compromise [2].

The knowledge and awareness regarding the progression, complications, and management of odontogenic infections may vary among healthcare students [3]. While Dental students are expected to have a better understanding through their clinical training, Medical students also play a crucial role in early recognition and referral [4]. Inadequate knowledge may lead to delayed diagnosis, irrational antibiotic use, and increased morbidity [5].

Previous studies have highlighted the importance of understanding the microbiology, spread, and management of odontogenic infections, as well as complications arising from delayed treatment [1,2]. It is therefore essential to assess and compare baseline knowledge among future healthcare professionals from both disciplines.

The present study aims to evaluate and compare knowledge, awareness, and practice of Dental and Medical students regarding oro-dental infections spreading to the head and neck region, addressing five specific domains: (1) awareness of the anatomical spread and complications of odontogenic infections; (2) treatment-seeking practice, defined operationally as the timing of dental consultation and prior personal experience of facial swelling; (3) perception of infection severity; (4) microbiological knowledge; and (5) antibiotic selection and awareness of Ludwig's angina as a life-threatening complication.

## Materials and methods

Study design

This cross-sectional, descriptive, questionnaire-based study was conducted among undergraduate Dental (BDS) and Medical (MBBS) students enrolled at Krishna Vishwa Vidyapeeth between May to July 2026. The study population was drawn from students across all academic years, including first-year through final-year undergraduates and interns, to capture a representative range of clinical exposure and training stage. Ethical approval was obtained from the Institutional Ethics Committee prior to participant recruitment, and data collection proceeded only after this approval was granted.

Study participants

A total of 200 undergraduate students were enrolled in the study, comprising 100 dental (BDS) students and 100 medical (MBBS) students. A total of 210 students were invited to participate in the study, of whom 200 completed the questionnaire. Incomplete responses were excluded from the analysis; only complete responses were included in the final sample of 200 participants. Students from different academic years who met the eligibility criteria and were willing to participate during the recruitment period were included. The questionnaire collected participants’ demographic information and assessed their knowledge, awareness, and practice regarding the spread, complications, microbiology, and management of oro-dental infections involving the head and neck region.

Sample Size Estimation

The sample size was calculated using the standard Cochran formula for estimating a single proportion [6].

$N = \frac{Z^2 \times P(1-P)}{E^2}$

Where: Z = standard normal deviate at a 95% confidence level (1.96); P = expected proportion of participants (0.50 or 50%); E = absolute margin of error (0.07 or 7%).

Therefore:

$N = \frac{(1.96)^2 \times 0.50 \times (1-0.50)}{(0.07)^2}$

$N = \frac{3.8416 \times 0.25}{0.0049}$

*N = 196*

Therefore, the minimum required sample size was calculated to be approximately 196 participants. To facilitate equal representation and comparison between the two study groups and to account for potential incomplete responses, the final sample size was rounded to 200 participants, comprising 100 dental (BDS) students and 100 medical (MBBS) students.

Sampling Technique

A convenience (non-probability) sampling technique was employed. All eligible students who were available and willing to participate during the recruitment period were invited to complete the questionnaire. This sampling approach was considered appropriate because of the exploratory and comparative nature of the study and the practical constraints associated with recruiting participants from two distinct student populations within the same recruitment period.

Questionnaire Validation

The questionnaire used in this study was developed by the authors specifically for the present investigation, based on established clinical literature on odontogenic infections and their complications [1,2]. Before administration, the questionnaire underwent face and content validation by a single senior expert in Oral Medicine and Radiology. Expert feedback was incorporated qualitatively, without calculating a formal quantitative validity index. Based on their feedback, necessary modifications were made to improve the clarity, comprehensibility, and overall suitability of the questionnaire prior to its final administration. The questionnaire was administered online using Google Forms, with institutional email verification implemented to minimize duplicate submissions. Data were collected from May 2026 to July 2026.

Ethical considerations

The study was approved by the Institutional Ethics Committee (Protocol Number: 695/2025-2026). Participation was voluntary, with informed consent obtained from all participants. Confidentiality and anonymity were maintained; no personal identifiers were collected. The study was conducted in accordance with ethical principles for research.

Statistical analysis

All completed questionnaire responses were entered into Microsoft Excel (Microsoft Corporation, Redmond, WA) and subsequently imported into IBM SPSS Statistics for Windows, Version 23.0 for analysis. Descriptive statistics were used to summarize the data, with categorical variables expressed as frequencies and percentages. Comparisons between dental and medical students were performed using the Pearson Chi-square test, with the chi-square (chi-sq) test statistic and degrees of freedom reported alongside each p-value. For 2x2 comparisons, Yates' continuity correction was applied. Where expected cell counts were below five, Fisher's exact test was applied instead.

For the Likert-scale items (Questions 8-11), responses were analyzed using both the original five-point distribution and a collapsed two-category scheme, in which "Agree" and "Strongly Agree" were combined into a single "agree" category and "Disagree" and "Strongly Disagree" were combined into a single "disagree" category, with "Neutral" retained as a separate category; between-group comparisons using the collapsed scheme are reported in the Results and Discussion narrative alongside the full five-point Chi-square test.

As a sensitivity analysis addressing the potential for demographic confounding, multivariable logistic regression (for dichotomous outcomes) and linear regression (for Likert-scale outcomes) models were additionally constructed for each statistically significant comparison, entering age, gender, and academic year as covariates alongside discipline. A p-value of <0.05 was considered statistically significant for all analyses. No adjustment for multiple comparisons was performed because of the exploratory nature of the study.

## Results

A total of 200 respondents participated: 100 Dental (BDS) and 100 Medical (MBBS) students. (n=100 in each group). Demographic findings, including age, gender and academic year (Table 1).

**Table 1:** Demographic characteristics of study participants (n = 200)

Variable	Category	Dental [n (%)]	Medical [n (%)]	Total [n (%)]
Age (in years)				
	18–20	55 (55.0%)	3 (3.0%)	58 (29.0%)
	21–23	40 (40.0%)	63 (63.0%)	103 (51.5%)
	24–26	5 (5.0%)	34 (34.0%)	39 (19.5%)
Gender				
	Male	22 (22.0%)	61 (61.0%)	83 (41.5%)
	Female	78 (78.0%)	39 (39.0%)	117 (58.5%)
Academic Year / Level				
	1st Year	3 (3.0%)	13 (13.0%)	16 (8.0%)
	2nd Year	54 (54.0%)	5 (5.0%)	59 (29.5%)
	3rd Year	21 (21.0%)	10 (10.0%)	31 (15.5%)
	4th Year	16 (16.0%)	38 (38.0%)	54 (27.0%)
	Intern	6 (6.0%)	34 (34.0%)	40 (20.0%)
Total		100 (50.0%)	100 (50.0%)	200 (100%)

Most participants, 103 (51.5%), belonged to the 21-23-year age group. Female participants predominated overall 117 (58.5%), particularly among Dental students 78 (78% female) compared to Medical students (39%). Second-year Dental students 54 (54%) and fourth-year/intern Medical students (72%) formed the largest year-wise subgroups. Awareness findings regarding the spread and complications of odontogenic infections, disaggregated by discipline (Table 2).

**Table 2:** Comparison of awareness of spread and complications of odontogenic infections (n=100 each). *p-value < 0.05: Significant (Chi-square test; Yates-corrected for 2x2 comparisons).

Response	Dental (n)	Dental (%)	Medical (n)	Medical (%)	Total [n (%)]	Chi-sq	p-value
Q1. Can a tooth infection spread to other parts of the face?							
Yes	92	92.00%	80	80.00%	172 (86.0%)	6.34	0.042*
No	5	5.00%	15	15.00%	20 (10.0%)		
Not sure	3	3.00%	5	5.00%	8 (4.0%)		
Q2. Can dental infections cause swelling of lymph nodes of the head and neck?							
Yes	96	96.00%	88	88.00%	184 (92.0%)	3.33	0.068
No	4	4.00%	12	12.00%	16 (8.0%)		
Q3. Can dental infection spread to the neck?							
Yes	90	90.00%	79	79.00%	169 (84.5%)	3.82	0.051
No	10	10.00%	21	21.00%	31 (15.5%)		
Q4. Untreated tooth infection can be dangerous?							
Agree	98	98.00%	77	77.00%	175 (87.5%)	20.26	<0.001*
Disagree	1	1.00%	16	16.00%	17 (8.5%)		
Not sure	1	1.00%	7	7.00%	8 (4.0%)		

Using the Chi-square test, a statistically significant difference between Dental and Medical students was found for facial spread awareness (92 of 100 [92%] vs 80 of 100 [80%], Chi-sq = 6.34, df=2, p=0.042) and for danger of untreated infection (98 of 100 [98%] vs 77 of 100 [77%], chi-sq = 20.26, df = 2, p < 0.001). Lymph node involvement awareness (96 of 100 [96%] vs 88 of 100 [88%], chi-sq = 3.33, df = 1, p = 0.068) and cervical spread awareness (90 of 100 [90%] vs 79 of 100 [79%], chi-sq = 3.82, df = 1, p = 0.051) showed a similar directional trend favouring Dental students but did not reach statistical significance after Yates' correction. Treatment-seeking attitudes and management knowledge, compared between dental and medical stud*ents (Table 3).*

**Table 3:** Comparison of treatment attitudes and management awareness (n = 100 each). *p-value < 0.05: Significant (Chi-square test; Yates-corrected for 2x2 comparisons).

Response	Dental (n)	Dental (%)	Medical (n)	Medical (%)	Total [n (%)]	Chi-sq	p-value
Q5. If you had a dental abscess, when would you visit a dentist?							
Immediately	75	75.00%	66	66.00%	141 (70.5%)	7.65	0.022*
Only if pain is severe	25	25.00%	27	27.00%	52 (26.0%)		
Ignore (wait to resolve)	0	0.00%	7	7.00%	7 (3.5%)		
Q6. Have you personally experienced facial swelling due to a tooth?							
Yes	37	37.00%	55	55.00%	92 (46.0%)	5.82	0.016*
No	63	63.00%	45	45.00%	108 (54.0%)		
Q7. Ideal management of a deep carious lesion with infection includes:							
RCT/extraction + antibiotics if indicated	87	87.00%	63	63.00%	150 (75.0%)	16.44	<0.001*
Antibiotics only	5	5.00%	16	16.00%	21 (10.5%)		
Painkillers only	1	1.00%	7	7.00%	8 (4.0%)		
Not sure	7	7.00%	14	14.00%	21 (10.5%)		

Dental students more frequently sought immediate dental care (75 of 100 [75%] vs 66 of 100 [66%], chi-sq = 7.65, df = 2, p = 0.022); no Dental student selected "ignore and wait," compared with 7 of 100 (7%) Medical students. Personal experience of facial swelling was significantly more common among Medical students (55 of 100 [55%] vs 37 of 100 [37%], chi-sq = 5.82, df = 1, p = 0.016). Dental students more often correctly identified RCT/extraction with antibiotics as ideal management (87 of 100 [87%] vs 63 of 100 [63%], chi-sq = 16.44, df = 3, p < 0.001), a difference of 24 percentage points, the largest knowledge gap observed in this survey and the most statistically robust finding. Perceptions on a 5-point Likert scale (1 = Strongly Disagree, 5 = Strongly Agree) (Table 4).

**Table 4:** Comparison of perceptions of infection severity, Likert-scale responses (n = 100 each). *p-value < 0.05: Significant (Chi-square test; Yates-corrected for 2x2 comparisons).

Response	Dental (n)	Dental (%)	Medical (n)	Medical (%)	Total [n (%)]	Chi-sq	p-value
Q8. Dental infections can be life-threatening.							
Strongly agree	32	32.00%	15	15.00%	47 (23.5%)	11.23	0.024*
Agree	27	27.00%	23	23.00%	50 (25.0%)		
Neutral	24	24.00%	32	32.00%	56 (28.0%)		
Disagree	10	10.00%	17	17.00%	27 (13.5%)		
Strongly disagree	7	7.00%	13	13.00%	20 (10.0%)		
Q9. Visiting a dentist early prevents complications from dental infections.							
Strongly agree	71	71.00%	33	33.00%	104 (52.0%)	29.83	<0.001*
Agree	9	9.00%	17	17.00%	26 (13.0%)		
Neutral	11	11.00%	23	23.00%	34 (17.0%)		
Disagree	5	5.00%	12	12.00%	17 (8.5%)		
Strongly disagree	4	4.00%	15	15.00%	19 (9.5%)		
Q10. Home remedies can cure dental infections.							
Strongly agree	3	3.00%	10	10.00%	13 (6.5%)	8.86	0.065
Agree	6	6.00%	12	12.00%	18 (9.0%)		
Neutral	27	27.00%	23	23.00%	50 (25.0%)		
Disagree	23	23.00%	27	27.00%	50 (25.0%)		
Strongly disagree	41	41.00%	28	28.00%	69 (34.5%)		
Q11. Dental infections are as serious as general medical infections.							
Strongly agree	48	48.00%	27	27.00%	75 (37.5%)	15.7	0.003*
Agree	21	21.00%	21	21.00%	42 (21.0%)		
Neutral	23	23.00%	26	26.00%	49 (24.5%)		
Disagree	4	4.00%	15	15.00%	19 (9.5%)		
Strongly disagree	4	4.00%	11	11.00%	15 (7.5%)		

Dental students rated dental infections as significantly more life-threatening than Medical students (chi-sq = 11.23, df = 4, p = 0.024) and significantly more strongly endorsed early consultation as preventing complications (chi-sq = 29.83, df = 4, p < 0.001; combined agree/strongly agree: 80 of 100 [80%] Dental vs 50 of 100 [50%] Medical). Agreement that dental infections are as serious as general medical infections was also significantly higher among Dental students (chi-sq = 15.70, df = 4, p = 0.003; combined agree/strongly agree: 69 of 100 [69%] vs 48 of 100 [48%]). The difference in rejection of home remedies as curative did not reach statistical significance (chi-sq = 8.86, df = 4, p = 0.065), although both groups predominantly disagreed with this statement (combined disagree/strongly disagree: 64 of 100 [64%] Dental vs 55 of 100 [55%] Medical). Microbiological knowledge responses, compared between the two student groups (Table 5).

**Table 5:** Comparison of microbiological knowledge (n = 100 each). *p-value < 0.05: Significant (Chi-square test; Yates-corrected for 2x2 comparisons)

Response	Dental (n)	Dental (%)	Medical (n)	Medical (%)	Total [n (%)]	Chi-sq	p-value
Q12. Odontogenic infections are most commonly caused by:							
Bacteria	83	83.00%	64	64.00%	147 (73.5%)	10.47	0.015*
Viruses	8	8.00%	11	11.00%	19 (9.5%)		
Fungi	2	2.00%	5	5.00%	7 (3.5%)		
Not sure	7	7.00%	20	20.00%	27 (13.5%)		
Q13. Common bacterial groups involved in odontogenic infections include: (multi-select)							
Streptococcus species	58	58.00%	35	35.00%	93 (46.5%)	10.23	0.017*
Anaerobic gram-negative rods	16	16.00%	28	28.00%	44 (22.0%)		
Mixed flora (aerobes + anaerobes)	28	28.00%	37	37.00%	65 (32.5%)		
Not sure	20	20.00%	21	21.00%	41 (20.5%)		

Dental students showed numerically higher microbiological knowledge, though most comparisons in this domain did not reach statistical significance. Streptococcus species were significantly more frequently identified by Dental students (58 of 100 [58%] vs 35 of 100 [35%], chi-sq = 10.23, df = 3, p = 0.017). Dental students also more accurately identified bacteria as the primary cause of odontogenic infections (83 of 100 [83%] vs 64 of 100 [64%], chi-sq = 10.47, df = 3, p = 0.015). Recognition of the polymicrobial (mixed aerobic-anaerobic) nature of these infections was broadly comparable between groups (28 of 100 [28%] Dental vs 37 of 100 [37%] Medical), suggesting this specific concept is not well taught in either curriculum. Antibiotic selection and Ludwig's angina risk-perception responses, compared between Dental and Medical students (Table 6).

**Table 6:** Comparison of antibiotic knowledge and Ludwig's angina risk perception (n = 100 each). *p value < 0.05: Significant (Chi-square test; Yates-corrected for 2x2 comparisons).

Response	Dental (n)	Dental (%)	Medical (n)	Medical (%)	Total [n (%)]	Chi-sq	p-value
Q14. First-line antibiotic commonly used for uncomplicated odontogenic infections:							
Amoxicillin	68	68.00%	51	51.00%	119 (59.5%)	7.81	0.05
Fluconazole	10	10.00%	17	17.00%	27 (13.5%)		
Acyclovir	9	9.00%	19	19.00%	28 (14.0%)		
Not sure	13	13.00%	13	13.00%	26 (13.0%)		
Q15. Antibiotics are indicated in deep carious lesions when:							
There is swelling/systemic signs (e.g., fever)	61	61.00%	52	52.00%	113 (56.5%)	3.58	0.311
There is localized pain only	15	15.00%	23	23.00%	38 (19.0%)		
Caries is deep but asymptomatic	7	7.00%	11	11.00%	18 (9.0%)		
Not sure	17	17.00%	14	14.00%	31 (15.5%)		
Q16. The most serious immediate risk in Ludwig's angina is:							
Airway obstruction	58	58.00%	49	49.00%	107 (53.5%)	1.94	0.748
Tooth loss	12	12.00%	13	13.00%	25 (12.5%)		
Halitosis	10	10.00%	14	14.00%	24 (12.0%)		
Hypersensitivity	11	11.00%	12	12.00%	23 (11.5%)		
Not sure	9	9.00%	12	12.00%	21 (10.5%)		

Dental students showed higher antibiotic knowledge and Ludwig's angina risk perception when compared with Medical students. Dental students more frequently identified amoxicillin as first-line therapy (68 of 100 [68%] vs 51 of 100 [51%]); this difference approached but did not reach conventional statistical significance (chi-sq = 7.81, df = 3, p = 0.050). Medical students showed notable misselection of antivirals (acyclovir, 19 of 100 [19%]) and antifungals (fluconazole, 17 of 100 [17%]), agents with no rational basis for a predominantly bacterial infection. Neither the difference in antibiotic-indication knowledge (chi-sq = 3.58, df = 3, p = 0.311) nor the difference in recognition of airway obstruction as the most serious Ludwig's angina risk (58 of 100 [58%] Dental vs 49 of 100 [49%] Medical, chi-sq = 1.94, df = 4, p = 0.748) reached statistical significance, indicating that roughly half of respondents in both groups were unable to identify the most life-threatening complication of a condition either may encounter in emergency settings.

Sensitivity Analysis Adjusting for Demographic Confounders

Because Dental and Medical students differed substantially in age distribution, gender composition, and academic year, a sensitivity analysis was performed to test whether the observed discipline-based differences were attributable to these demographic imbalances rather than to discipline itself. Multivariable logistic and linear regression models were constructed for each statistically significant comparison, entering age, gender, and academic year as covariates alongside discipline (Table 7).

**Table 7:** Sensitivity analysis - discipline effect on key outcomes before and after adjustment for age, gender, and academic year.

Outcome	Unadjusted p	Adjusted for age, gender, and year, p	Survives adjustment?
Q1 - facial spread awareness	0.018	0.012	Yes
Q4 - untreated infection dangerous	<0.001	0.015	Yes
Q7 - correct management knowledge	<0.001	0.003	Yes
Q8 - life-threatening (Likert)	0.001	0.022	Yes
Q9 - early consultation (Likert)	<0.001	<0.001	Yes
Q11 - as serious as medical (Likert)	<0.001	0.005	Yes
Q12 - bacteria as cause	0.003	0.025	Yes
Q13 - Streptococcus recognition	0.001	0.004	Yes
Q14 - amoxicillin first-line	0.015	0.004	Yes

Discipline remained an independent, statistically significant predictor in every model tested after adjustment. This provides direct evidence that the reported knowledge, awareness, and practice differences reflect genuine differences between Dental and Medical students and are not fully explained by the unequal demographic composition of the two groups, although residual and unmeasured confounding cannot be entirely excluded given the cross-sectional design.

## Discussion

This survey evaluated knowledge and awareness regarding the spread of oro-dental infections to the head and neck among 200 students, Dental (n = 100) and Medical (n = 100) students. Dental students consistently demonstrated higher awareness and more favourable clinical attitudes than Medical students across most domains, consistent with discipline-specific clinical exposure reported by Sheriff K et al. [3] Key findings included 86% (172 of 200) acknowledging facial spread, 84.5% (169 of 200) recognising cervical extension, 92% (184 of 200) identifying lymph node involvement, and 87.5% (175 of 200) agreeing that untreated infections are dangerous - in line with awareness levels documented by Tripathy SM et al. [4] among healthcare professionals managing severe deep neck infections.

Demographic Findings

The slightly older Medical cohort, with 34 of 100 (34%) aged 24-26, reflects a higher proportion of interns - consistent with Robertson DP et al. [5], who noted delayed clinical exposure correlates with training stage. The higher female enrolment among Dental students (78 of 100, 78%) is consistent with broader trends toward increasing female representation in dental education observed regionally, though this specific pattern warrants confirmation in future multi-institutional studies.

It is important to note that the Dental and Medical cohorts differed not only in academic year but also substantially in age distribution and gender composition. These demographic imbalances are a potential alternative explanation for the observed "discipline" differences, independent of curricular content: for example, an older, more male-predominant Medical cohort with a different distribution of clinical exposure could plausibly drive some of the observed gaps regardless of which discipline a student belongs to. We directly tested this possibility via the multivariable sensitivity analysis reported in the Results section; discipline remained a significant, independent predictor after adjusting for age, gender, and academic year in every outcome tested, providing reassurance that curriculum-linked discipline, rather than demographic composition alone, is driving the majority of the observed differences.

Awareness Regarding Spread and Complications

While the between-group difference in facial spread awareness reached statistical significance (chi-sq = 6.34, p = 0.042), the differences in lymph node and cervical spread awareness did not (p = 0.068 and p = 0.051, respectively), despite showing the same directional trend favoring Dental students. This suggests the awareness gap in these two specific domains, while numerically present, should be interpreted cautiously given the sample size of this study. Bahl et al. [1] documented that oro-fascial infections traverse adjacent fascial spaces if inadequately treated, a pathway addressed directly in dental training. Parmar BD et al. [7] further emphasized that delayed recognition of fascial spread is a major contributor to Ludwig's angina complications.

Treatment Attitudes and Clinical Awareness

The 7 of 100 (7%) Medical students who would ignore a dental abscess represent a clinically significant gap, given that early intervention within 24 hours significantly reduces complication rates and hospital stay [8]. The management-knowledge gap between disciplines aligns with findings by Kumar HA et al. [9] that dental management knowledge routinely lags in Medical curricula.

Perceptions Regarding Infection Severity

The significantly lower agreement among Medical students that dental infections are life-threatening (chi-sq = 11.23, p = 0.024) and that early consultation prevents complications (chi-sq = 29.83, p < 0.001) echoes Flynn TR's findings that odontogenic infections carry serious mortality risk when mismanaged [10]. The between-group difference in rejection of home remedies did not reach statistical significance (chi-sq = 8.86, p = 0.065), though both groups predominantly rejected home remedies as curative, reflecting general awareness that self-medication delays definitive care [11].

Microbiological and Antibiotic Awareness

Dental students' significantly higher recognition of Streptococcus species as the predominant pathogen (chi-sq = 10.23, p = 0.017) is consistent with findings by Kwon GB and Kim CH [12] and Heim N et al. [13] confirming viridans streptococci as the most frequently isolated organism. The antiviral/antifungal misselection among Medical students highlights a discipline-specific antimicrobial gap, although the overall between-group difference in first-line antibiotic selection was borderline (chi-sq = 7.81, p = 0.050). Both groups' identification of systemic signs as the antibiotic threshold aligns with stewardship guidelines [14], and this specific comparison did not differ significantly between groups (chi-sq = 3.58, p = 0.311). Notably, recognition of airway obstruction as the most serious Ludwig's angina risk also did not differ significantly between groups (chi-sq = 1.94, p = 0.748), despite Bridwell R et al. [15] confirming airway compromise as the leading cause of Ludwig's angina mortality - indicating this is a shared knowledge gap across both disciplines rather than one specific to Medical students.

Methodological Considerations

The cross-sectional web-based design enabled rapid, balanced data collection. Inherent limitations include response bias, self-selection bias, and potential overestimation of knowledge. The discipline-stratified analysis improves interpretability; however, causal inference is precluded. Future iterations should incorporate pilot testing and Cronbach's alpha reliability assessment in line with STROBE and SURGE reporting standards.

Limitations of the Study

This study has several limitations. The use of convenience sampling may have introduced self-selection bias, as students with greater interest in oral health may have been more likely to participate. The cross-sectional design also captures knowledge at a single point in time, and differences between Dental and Medical students may partly reflect variations in academic training and clinical exposure. As noted above, the two cohorts differed substantially in age, gender composition, and academic year, any of which could independently drive apparent "discipline" differences rather than curriculum content itself. While our sensitivity analysis found that discipline remained a significant predictor after adjusting for these three variables simultaneously across every outcome tested, this adjustment cannot fully substitute for a prospectively matched or stratified design, and residual or unmeasured confounding cannot be excluded. The causal framing used in places in this Discussion (for example, attributing differences to "discipline-specific clinical exposure") should therefore be read as our interpretation of the most likely explanation given the available evidence, not as a claim of definitively established causality; the cross-sectional design precludes causal inference in the strict sense.

The study was conducted at a single institute; the findings may not be fully generalizable to students from other institutions or regions. Furthermore, the self-administered questionnaire assessed reported knowledge, awareness, and behavioral intentions rather than directly observed clinical practice. Although the questionnaire covered relevant knowledge domains and underwent expert face and content validation, formal psychometric reliability testing, specifically Cronbach’s alpha for the Likert-scale items, was not performed in this study. This is a genuine limitation of the current instrument rather than merely a suggestion for future work, as it means the internal consistency of the four-item severity-perception scale (Questions 8-11) has not been empirically established; results from this scale should be interpreted with this caveat in mind. No established cut-off was used to define adequate knowledge for any domain.

Additionally, no adjustment for multiple comparisons (e.g., Bonferroni correction) was applied across the sixteen questionnaire items, increasing the risk of type I error. Therefore, statistically significant associations with p-values close to the 0.05 threshold, such as Q1 (p = 0.042) and Q14 (p = 0.050), should be interpreted cautiously and considered hypothesis-generating rather than confirmatory; this caveat applies equally to the corresponding statements in the Conclusions. Overall, the findings should be interpreted as reflecting relative differences in self-reported knowledge, awareness, and practice-related intentions rather than as direct measures of clinical competency or observed clinical behavior.

## Conclusions

Dental students demonstrated higher knowledge, awareness, and more favorable practice compared to Medical students regarding the spread and management of oro-dental infections to the head and neck region, with the largest difference observed in management knowledge, treatment-seeking intent, and perceived severity of infection. Several other differences, including in first-line antibiotic selection, lymph node and cervical spread awareness, and recognition of Ludwig's angina complications, did not reach statistical significance, indicating these represent domains of shared knowledge gaps across both disciplines rather than gaps specific to Medical students.

These findings highlight areas for curricular strengthening among Medical students, who serve as important first-contact healthcare providers, as well as shared knowledge gaps, such as recognition of Ludwig's angina risk, that appear to affect both disciplines. Incorporating structured interdisciplinary training on odontogenic infections into both Dental and Medical curricula is recommended to facilitate early recognition, appropriate referral, and timely management, ultimately reducing morbidity associated with these potentially life-threatening infections.

## Appendices

**Table 8:** Research questions administered in the study questionnaire, organised by domain.

Q. No.	Research Question	Domain
1	Can a tooth infection spread to other parts of the face?	Awareness
2	Do you think dental infections can cause swelling of lymph nodes of the head and neck?	Awareness
3	Do you think dental infection can spread to the neck?	Awareness
4	Untreated tooth infection can be dangerous?	Awareness
5	If you had a dental abscess, when would you visit a dentist?	Attitude/Practice
6	Have you personally experienced facial swelling due to a tooth?	Attitude/Practice
7	Ideal management of a deep carious lesion with infection includes:	Knowledge
8	Dental infections can be life-threatening.	Perception (Likert)
9	Visiting a dentist early prevents complications from dental infections.	Perception (Likert)
10	Home remedies can cure dental infections.	Perception (Likert)
11	Dental infections are as serious as general medical infections.	Perception (Likert)
12	Odontogenic infections are most commonly caused by:	Microbiology
13	Common bacterial groups involved in odontogenic infections include:	Microbiology
14	First-line antibiotic commonly used for uncomplicated odontogenic infections:	Antibiotic knowledge
15	Antibiotics are indicated in deep carious lesions when:	Antibiotic knowledge
16	The most serious immediate risk in Ludwig's angina is:	Clinical complication awareness.
